# Concave Urinary Crystallines: Direct Evidence of Calcium Oxalate Crystals Dissolution by Citrate *In Vivo*

**DOI:** 10.1155/2013/637617

**Published:** 2013-11-18

**Authors:** Yun-Feng Shang, Meng Xu, Guang-Na Zhang, Jian-Ming Ouyang

**Affiliations:** ^1^Yueyang Occupation Technical College, Yueyang, Hunan 414000, China; ^2^Institute of Biomineralization and Lithiasis Research, Jinan University, Guangzhou 510632, China

## Abstract

The changes in urinary crystal properties in patients with calcium oxalate (CaOx) calculi after oral administration of potassium citrate (K_3_cit) were investigated via atomic force microscopy (AFM), scanning electron microscopy (SEM), X-ray powder diffractometry (XRD), and zeta potential analyzer. The AFM and SEM results showed that the surface of urinary crystals became concave, the edges and corners of crystals became blunt, the average size of urinary crystallines decreased significantly, and aggregation of urinary crystals was reduced. These changes were attributed to the significant increase in concentration of excreted citrate to 492 ± 118 mg/L after K_3_cit intake from 289 ± 83 mg/L before K_3_cit intake. After the amount of urinary citrate was increased, it complexed with Ca^2+^ ions on urinary crystals, which dissolved these crystals. Thus, the appearance of concave urinary crystals was a direct evidence of CaOx dissolution by citrate *in vivo*. The XRD results showed that the quantities and species of urinary crystals decreased after K_3_cit intake. The mechanism of inhibition of formation of CaOx stones by K_3_cit was possibly due to the complexation of Ca^2+^ with citrate, increase in urine pH, concentration of urinary inhibitor glycosaminoglycans (GAGs), and the absolute value of zeta potential after K_3_cit intake.

## 1. Introduction

Nephrolithiasis, characterized by renal calculi, is a disorder that has recently shown a trend of rising incidence around the world. Renal calculi mainly contain calcium oxalate (CaOx) crystals [1]. However, knowledge about its pathogenesis is limited thus far [2, 3], and the high recurrence rate is still an important clinical issue [4].

Although CaOx is supersaturated in urine, the formation of renal calculi in healthy people is difficult because of all kinds of inhibitors in urine, including citrate, magnesium, osteopontin, and tyrosine hydroxylase [5]. Chemically speaking, CaOx calculi formation is closely related to with the following factors: high concentration of calcium and oxalate in urine, nucleation, growth and aggregation of CaOx crystals, and adhesion of calcium oxalate monohydrate (COM) to renal tubular epithelial cells [6]. CaOx calculi formation is thought to follow two main routes, (slow) formation of subepithelial plaques at papillary tips and (rapid) formation of intratubular plugs [7]. 

Potassium citrate (K_3_cit) is one of the main drugs for treatment and prevention of renal calculi. In July 1985, the US Food and Drug Administration approved K_3_cit as a single drug to treat CaOx calculi with low urinary citrate and uric acid calculi. As a clinical drug, K_3_cit is widely used for its advantages such as nontoxicity, low price, few side effects, and potential for long-term use. Studies have shown that after K_3_cit intake in patients with chronic renal calculi, the occurrence rate of new renal calculi was one-fifth of that without K_3_cit intake [8]. When patients with renal calculi were orally administered K_3_cit after treatment of extracorporeal shock wave lithotripsy, the recurrence rate of renal calculi was 0 in 12 months, whereas the recurrence rate of patients without K_3_cit intake was 28.5% (*P* < 0.05) [9]. Therefore, understanding the mechanism of K_3_cit has a significant scientific and practical application for the prevention and treatment of renal calculi. 

However, little is known about the change in urinary crystal properties in patients with CaOx calculi after K_3_cit administration. After ten years of urinary crystal study, we found that after K_3_cit intake, crystal depressions emerge on the surfaces of some urinary crystals in patients with CaOx calculi, which is direct evidence that citrate dissolves CaOx calculi *in vivo*. Based on this previous finding, this study examines the crystal depression that emerges on the surfaces of some urinary crystals, especially via atomic force microscope (AFM). The changes in urine citrate and urine GAGs as well as pH and zeta potential were detected, and the formation mechanism of concave crystals was discussed. Our results can shed light on the exploration of preventing renal calculi formation from a chemical perspective.

## 2. Experiments

### 2.1. Reagents and Apparatus

Sodium azide (NaN_3_) and all other reagents used were all analytically pure. All the glassware was washed clean with secondary distilled water.

Urine crystals were observed using a Philips XL-30 environmental scanning electron microscope (SEM) at 20 kV after being covered by an ultrathin layer of gold. Atomic force microscopy (AFM) was performed in contact mode with air by using commercial AFM (AutoProbe CP, ThermoMicroscopes, USA). Microfabricated silicon nitrite cantilevers (Park Scientific Instruments) were used. The 512 × 512 pixel AFM images were obtained using the software (ThermoMicroscopes Proscan Image Processing Software Version 2.1) provided with the instrument, which can eliminate low-frequency background noise in the scanning direction. X-ray diffraction (XRD) results were obtained using a D/max-*γ*A X-ray diffractometer (Rigaku, Japan) by using Ni-filtered Cu-K_*α*_ radiation (*λ* = 1.54 Å) at a scanning rate of 2° min^−1^ and a scanning range (2*θ*) from 5° to 60°. The zeta potential was determined using a nanoparticle size analyzer from Zetasizer Nano-ZS (Malvern, England) in the following testing conditions: incident beam: He-Ne laser (*λ* = 633.0 nm) incident angle: 90°; temperature: 25.0 ± 0.1°C. pH values were measured using a PHS-3C precision pH meter (Shanghai Precision Scientific Instrument Co. Ltd.).

### 2.2. Collection and Treatment of Stones and Component Characterization

The participants in the study included 30 randomly selected lithogenic patients (18 men and 12 women; mean age = 53.1 years; range = 21~73 years; all of them were from the Lithotripsy Center of the First Affiliated Hospital of Jinan University) and 30 randomly selected healthy humans with no prior history of urinary stones (16 men and 14 women; mean age = 37.5 years; range = 22~56 years; all of them were from the graduates and teachers of Jinan University). Urinary stones were collected after surgery, disinfected with 75% alcohol (A.R. grade), rinsed with distilled water, and placed in a dust-free incubator at 40°C to dry. The urinary stones were then ground into powder by an agate mortar for X-ray diffraction (XRD) characterization, which showed that the quality fraction of CaOx in stones was between 80% and 100% and that the stones contained small amounts of calcium phosphate and uric acid.

### 2.3. Collection, Treatment, and Detection of Urine

The changes in urinary crystal property in 30 patients (from the same 30 patients above) before and after K_3_cit intake were studied for a week, and the dose of K_3_cit (in tablet) was set at 2.538 g/d. None of the patients had gastric intolerance.

Urine treatment and urinary crystallite collection were carried out according to the methods reported in the literature [10–14]. Fasting morning urine samples were collected. After the pH value was detected, 2% NaN_3_ solution (10 mL/L urine sample) was added into the urine samples as an antiseptic, and zeta potential measurements were taken. Subsequently, anhydrous alcohol was added into the urine sample (urine : ethanol = 3 : 2); then the urine was stirred and left undisturbed for half an hour to make proteins denaturalize and deposit. The supernatant was directly used to detect the micron-sized crystals in urine by means of XRD, AFM, and SEM.

*XRD Detection*. About 50 *μ*L of the urine sample was placed on 12 mm × 12 mm clean glass slides by using a microsyringe. The glass slides were then dried in an oven at 50 ± 5°C for 2 h to volatilize the urine. This process was repeated four times. The urinary crystals were then treated according to a previously described method [15] to remove the soluble fractions of sodium chloride and urea. That is, the glass slides with urinary crystals were slowly immersed in double distilled water at a 45° angle of tilt and gently shaken for 1 min, and the soluble fractions (such as sodium chloride and urea) precipitated from urine during drying process could be removed. After carefully taking out the glass slides, water from the edge of the slides was dried using an absorbent paper and the slides were again dried in a vacuum desiccator for 1 d to make samples that could be used for XRD characterization.

*SEM and AFM Detection*. About 30 *μ*L of the urine sample was placed on 10 mm × 10 mm clean glass slides or a clean mica sheet by using a microsyringe. The glass slides and the mica sheet were then dried in a vacuum dryer. Then a similar washing process as above was carried out to remove the soluble crystallites. 

*Zeta Potential Detection*. Urine samples were directly processed for zeta potential detection by using a Zetasizer Nano-ZS nanoparticle size analyzer.

### 2.4. Detection of Citrate and Glycosaminoglycans (GAGs) Excretion Amount before and after K_3_cit Intake

Ammonium metavanadate-assisted catalytic-kinetic spectrophotometry was performed to determine urinary citrate content [16], and Alcian Blue colorimetric method was used to determine urinary GAGs content [17]. 

## 3. Results

### 3.1. SEM Data of Urinary Crystals before and after K_3_cit Intake

**Figure 1:**
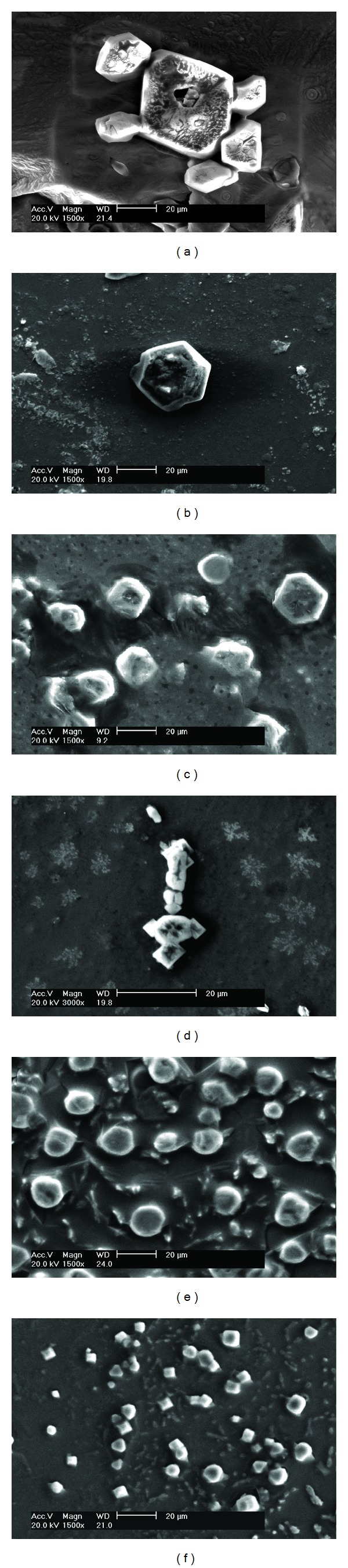
SEM images of representative urinary crystals from patients with CaOx renal calculi after oral administration of K_3_cit for one week. Scale bar: 20 *μ*m.

Figure 1 shows the SEM images of the urinary crystals in patients with renal calculi after K_3_cit intake for a week. Crystal depressions clearly emerged on the surface of some crystals in the urine (Figures 1(a) to 1(d)). By contrast, these depressions rarely occur in urinary crystals before K_3_cit intake. About one-third of the urinary crystals from the 30 patients with CaOx calculi were concave after K_3_cit intake.

Aside from crystal depressions that emerged on the surface of crystals, the morphology of urinary crystals showed other changes, such as the rounding and smoothing of the corners and edges (Figure 1(e)). In addition, the size of the crystals significantly decreased, the proportion of calcium oxalate dihydrate (COD) with tetragonal bipyramid increased (Figure 1(f)), and the aggregation of the crystals decreased.

### 3.2. AFM Data of Urinary Crystals before and after K_3_cit Intake

**Figure 2:**
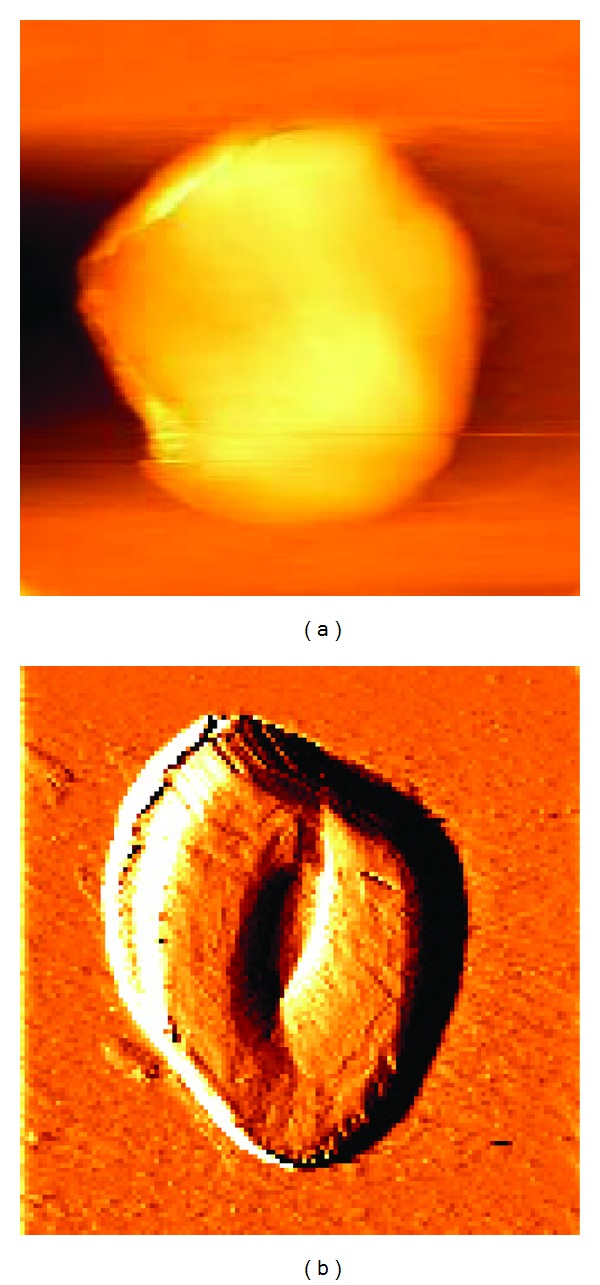
AFM images of urinary crystal of one patient with CaOx calculi before (a) and after (b) K_3_cit intake. Image size: 3 *μ*m × 3 *μ*m.

To further study these concave crystals, the morphology was observed via AFM.  Figure 2 shows AFM images of urinary crystals of a patient with CaOx calculi before and after K_3_cit intake for a week, and the results show the crystal depressions.

### 3.3. XRD Detection of Urinary Crystals before and after K_3_cit Intake

**Figure 3:**
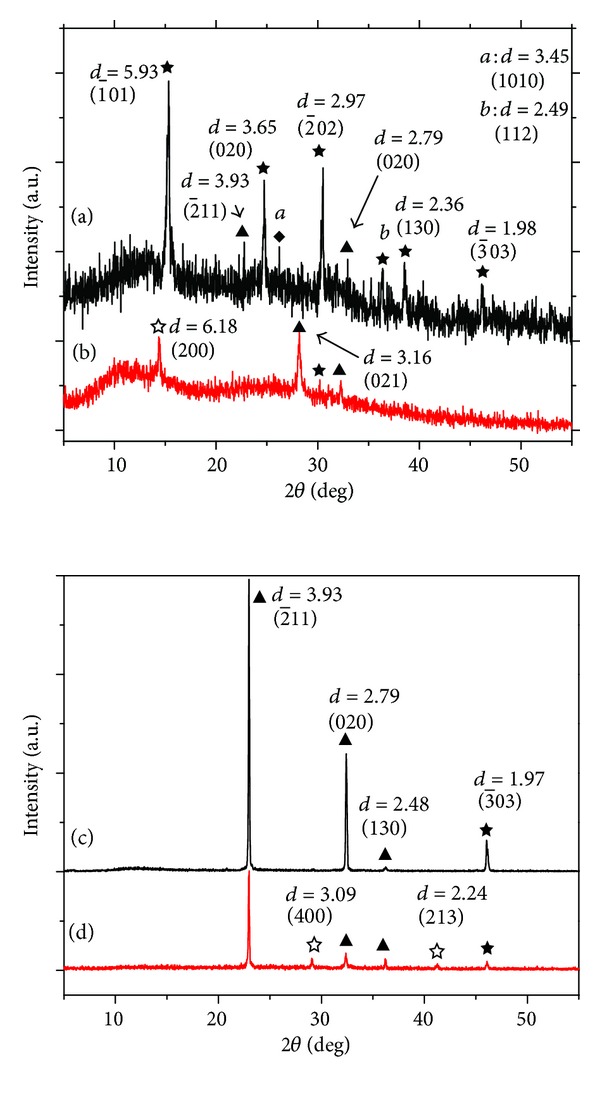
XRD patterns of urinary crystals of two patients with CaOx calculi before ((a), (c)) and after ((b), (d)) K_3_cit intake. ★: COM; ☆: COD; ▲: uric acid; ◆: *β*-Ca_3_(PO_4_)_2_.

Figure 3 shows the XRD patterns of urinary crystals in two patients with CaOx calculi before and after K_3_cit intake. The number of diffraction peaks significantly decreased after K_3_cit intake, indicating that the species of urinary crystals decrease after K_3_cit intake. The intensity of diffraction peaks also significantly decreased, which was approximately one-third of that before K_3_cit intake. This result indicated that the quality of urinary crystals decreased after K_3_cit intake. The proportion of COM in the urine decreased after K_3_cit intake, whereas that of COD increased. In detail, compared with the XRD patterns before K_3_cit intake (Figures 3(a) and 3(c)), the diffraction peaks of COM at *d* = 5.93, 3.65, 2.97, 2.36, and 1.98 Å disappeared (Figure 3(b)) [18], whereas the diffraction peaks of COD at *d* = 6.18 Å (Figure 3(b)) or 3.09 and 2.24 Å (Figure 3(d)) appeared, which showed that the amount of COM crystals significantly decreased after K_3_cit intake, whereas the relative amount of COD increased. This result was consistent with that of the SEM data (Figure 1(f)). Moreover, the diffraction peaks attributed to uric acid and calcium phosphate disappeared or significantly weakened after K_3_cit intake.

Among the 30 stone patients, 21 patients (70%) had crystalluria, in which 10 patients had COM as main composition, 1 patient had COD as main composition, and 2 patients had both COM and COD. The other compositions were uric acid (UA, seven cases) and calcium phosphate (CaP, one case). However, after K_3_cit intake, only seven patients had still crystalluria, in which 1 patient had COM, 2 patients had COD, and 4 patients had both COM and COD. The other composition was urate, uric acid, and CaP.

### 3.4. Concentration Differences of Citrate and GAGs in Urine before and after K_3_cit Intake

**Figure 4:**
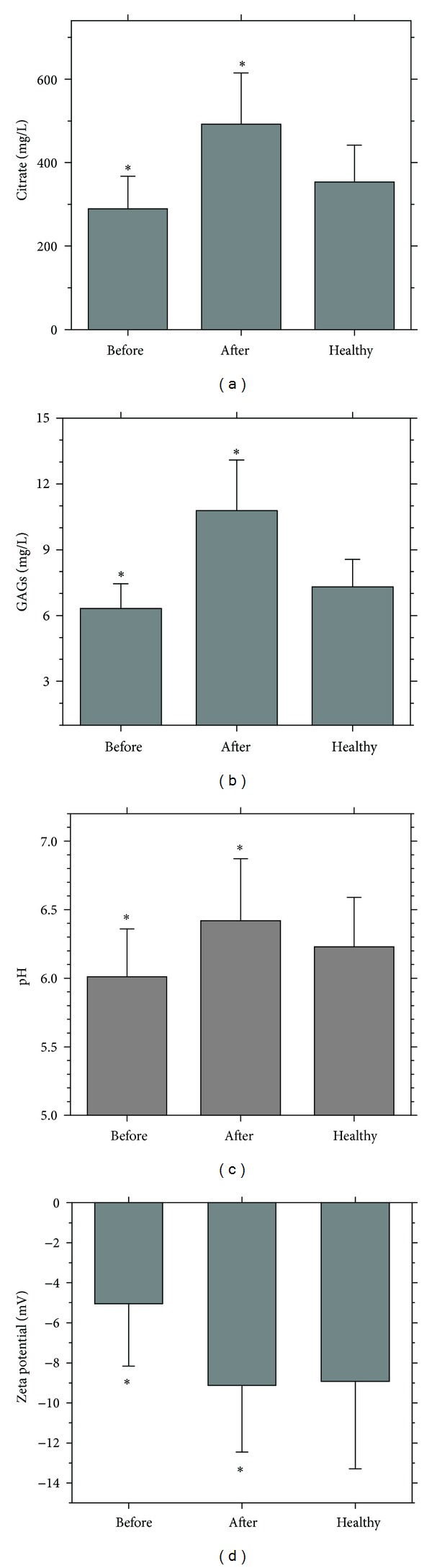
Comparison between the properties of urine and urinary crystals from healthy control individuals and patients with CaOx calculi before and after K_3_cit intake. (a) Excretion amount of citrate; (b) excretion amount of GAGs; (c) urinary pH; (d) zeta potential (*n* = 30).

Before K_3_cit intake, the citrate content in urine of patients with CaOx calculi was 289 ± 83 mg/L (Figure 4(a)) and increased to 492 ± 118 mg/L (*P* < 0.01) after K_3_cit intake, whereas that of the control sample was 354 ± 97 mg/L. After K_3_cit intake, GAGs increased to 10.78 ± 2.31 mg/L (*P* < 0.01) from 6.32 ± 1.13 mg/L before K_3_cit intake (Figure 4(b)), whereas that of the control sample was 7.30 ± 1.26 mg/L. Urine pH increased to 6.42 ± 0.45 from 6.01 ± 0.35 before K_3_cit intake, whereas that of the control sample was 6.23 ± 0.36 mg/L (Figure 4(c)).

The obtained values were consistent with those of previous studies. For example, Trinchieri et al. [19] found that the excretion amounts of citrate in urine of patients with COM and COD calculi were 507 ± 264 and 487 ± 247 mg/24 h, respectively (24 h urine volume in healthy control individuals was about 1.8 L to 2 L, whereas the value was 1.2 L in patients with renal calculi). Srinivasan et al. [20] found that the excretion amount of citrate in urine of healthy control individuals (601 ± 84 mg/24 h) was higher than that of uric acid (UA), CaOx, and calcium phosphate (CaP) calculi, which were 415 ± 43, 429 ± 58, and 492 ± 52 mg/24 h, respectively. Gu et al. [21] randomly divided 252 patients with CaOx calculi into two groups. For the group after K_3_cit intake for one week, the average urine pH increased to 6.81 from 5.88 (*P* < 0.001) and urinary citrate content increased to 1.96 from 0.78 mmol/24 h (*P* < 0.001), whereas urinary calcium content decreased to 4.44 from 7.81 mmol/24 h.

### 3.5. Zeta Potential on the Surface of Urinary Crystallines before and after K_3_cit Intake

Before K_3_cit intake, the zeta potential of urinary crystals in patients with renal calculi was −5.05 ± 3.12 mV and became −9.13 ± 3.33 mV (*P* < 0.05) after K_3_cit intake, whereas the zeta potential of urinary crystals in healthy control individuals was −8.93 ± 4.36 mV (Figure 4(d)).

## 4. Discussion

### 4.1. Appearance of Concave Crystals and Rounded Crystals after K_3_cit Intake

Whether it was the group which had hypocitraturia or the group with normocitraturia, the citrate content in urine of patients increased after K_3_cit intake. But there was no linear relationship between the increment of citrate excretion and the amount of urine citrate before K_3_cit intake. The concave urinary crystals appeared in the urine with a high citrate excretion amount after K_3_cit intake.

Citrate is one of the most abundant anions in urine and an important inhibitor of calcium calculi [22]. An excretion amount of citrate less than 320 mg/24 h (about 267 mg/L) is defined as hypocitraturia, which indicates a risk of stone formation [23].

Figure 4(a) shows that the citrate excretion in urine after K_3_cit intake for a week increased to 492 ± 118 mg/L from 289 ± 83 mg/L before K_3_cit intake. Before K_3_cit intake, 14 cases of the 30 stone patients studied had urinary citrate level lower than 267 mg/L. After K_3_cit intake, none of them had urinary citrate level lower than 267 mg/L (the minimum value was 270 mg/L). For the healthy controls, still 6 cases of the 30 persons studied had urinary citrate level lower than 267 mg/L.

When citrate excretion increases, the formation of CaOx calculi is possibly inhibited through the following mechanisms. Citric acid is a tricarboxylic acid with acid dissociation constants (pKa) of 2.9, 4.3, and 5.6 [24]. Citrate can complex with Ca^2+^ ions to form a chelate with a five- or six-membered ring, with a stability constant of 4.79 × 10^4^ [24] and excellent solubility. Thus, increasing citrate excretion in urine can chelate citrate with Ca^2+^ ions on the surface of CaOx crystals. This chelation slowly dissolves the crystals, which leads to the emergence of concave crystals (Figures 1(a) to 1(d)). Therefore, the emergence of concave crystals is direct proof that citrate chelates with Ca^2+^ ion to dissolve CaOx crystals, thereby inhibiting CaOx calculi formation.

Nine patients of the 30 stone patients studied had urinary crystallites with concave surface after K_3_cit intake. Their citrate excretion amounts were in the region from 510 to 725 mg/L with an average of 631 mg/L, which was higher than the average value of citrate excretion amounts after K_3_cit intake (492 mg/L).

### 4.2. Inhibition of K_3_cit on CaOx Calculi Formation

(1) K_3_cit makes the tips of the crystals round and smooth.

Citrate can maintain a complexation-dissociation equilibrium with the Ca^2+^ ions on the surface of CaOx crystals in urine, especially those surrounding and on the tips of CaOx crystals. These crystals are continuously dissolved because of the complexation with citrate. At the same time, the dissolved Ca^2+^ ions are continuously deposited on the surface of CaOx crystals. This continuous dissolution-deposition caused the morphology of crystals to be more rounded and blunt after K_3_cit intake (Figure 1(e)).

(2) K_3_cit inhibits aggregation of urinary crystals.

After the tips and edges of crystals became round and smooth, the tendency of crystal aggregation was reduced. In addition, the increase in zeta potential absolute value on the surface of urinary crystals after K_3_cit intake (Figure 4(d)) was also favorable to the increase in repulsive force between crystals and inhibited the aggregation of urinary crystals. Inhibition of aggregation will affect particle size and thus may prevent plug formation.

(3) K_3_cit can decrease the size of urinary crystals. 

The excretion amounts of citrate (Figure 4(a)) and glycosaminoglycans (GAGs) (Figure 4(b)) in urine increased after K_3_cit intake. Both citrate and GAGs can chelate with Ca^2+^ ions; citrate chelates with Ca^2+^ to form 1 : 1 complexes, and 1 *μ*mol disaccharide unit of chondroitin sulfate (one of the GAGs) can complex with 0.757 *μ*mol Ca^2+^ [25]. These chelated compounds were soluble; thus, the Ca^2+^ concentration and CaOx saturation in urine decreased, and the growth of CaOx crystals was inhibited. Therefore, the size of urinary crystals decreased after K_3_cit intake.

(4) In addition, citrate is active in the nucleation phase of calcium oxalate and has a profound effect on the morphology of the crystals formed [26]. It retards nucleation at high concentration. Therefore, it can explain the reduced number of crystals in Figure 1. 

(5) K_3_cit inhibits the formation of UA crystals and reduces the heterogeneous nucleation process of COM crystals. A lot of UA crystals can be found among urinary crystals. The XRD spectra (Figure 3) show that UA quality significantly decreased after K_3_cit intake. For example, compared with the XRD pattern before K_3_cit intake, the diffraction peaks assigned to the ($\overset{-}{2}$11) and (020) faces of UA at *d* = 3.93 and 2.79 Å significantly weakened. Thus, the quality of UA crystallites in urine remarkably decreased. This result was attributed to the excreted citrate in urine, which alkalinized the urine (Figure 4(c)). Thus, urine pH increased. Moreover, most of the UA crystals were turned into urate, a much more soluble species, thereby decreasing the UA content in urine. After the amount of UA crystals in urine decreased, the heterogeneous nucleation process of COM induced by UA crystals also decreased, thereby inhibiting the formation of CaOx calculi. 

(6) K_3_cit converts COM crystals to COD crystals. The SEM data (Figure 1(f)) and XRD analysis (Figures 3(a), and 3(b)) show that the content of COM in urinary crystals before K_3_cit intake was higher than that after K_3_cit intake, whereas the content of COD after K_3_cit intake was significantly higher than that before K_3_cit intake. This result was attributed to the high affinity of citrate to COM, which largely inhibited the development of COM [27–29]. Kok [26] reported that citrate steered crystal morphology from the coffin-shaped classic COM through more rounded COM crystals and finally converted to exclusively COD crystals. Wierzbicki et al. [30] studied the bonding of citrate with COM crystals by using a molecular model and calculated the bonding energy of citrate with different COM crystal faces, thereby indicating that citrate could better coordinate with Ca^2+^ on a ($\overset{-}{1}01$) plane of COM. CaOx calculi mainly existed in the form of COM and COD, but COD was easier to evacuate from the body in urine than COM [31]. Therefore, if more COD crystals can be induced or the transformation of COM to COD can be promoted, renal calculi formation can be prevented and its relapse can be delayed.

## 5. Conclusions

Urinary crystal depressions were first observed in patients with CaOx calculi after K_3_cit intake, and these depressions are direct proof of the dissolution of CaOx crystals by citrate, thereby preventing CaOx calculi formation. The cause of crystal depression formation was explained. After K_3_cit intake for one week, the aggregation of urinary crystals in patients with CaOx calculi significantly decreased, the species and amount of the crystals decreased, COD content increased, and COM content decreased. In addition, the zeta potential absolute value on the crystal surface increased because of the increase in urinary citrate and GAGs excretion amount as well as urine alkalization. These results inhibit CaOx calculi formation. The results in this work will help shed light on the prevention of kidney stones formation and the delay of its recurrence.
